# Protein Synthesis Inhibition Activity by Strawberry Tissue Protein Extracts during Plant Life Cycle and under Biotic and Abiotic Stresses

**DOI:** 10.3390/ijms140815532

**Published:** 2013-07-25

**Authors:** Letizia Polito, Massimo Bortolotti, Daniele Mercatelli, Rossella Mancuso, Gianluca Baruzzi, Walther Faedi, Andrea Bolognesi

**Affiliations:** 1 Department of Experimental, Diagnostic and Specialty Medicine (DIMES); Alma Mater Studiorum-University of Bologna, Bologna 40126, Italy; E-Mails: letizia.polito@unibo.it (L.P.); massimo.bortolotti2@unibo.it (M.B.); daniele.mercatelli2@unibo.it (D.M.); ros_mancuso@yahoo.it (R.M.); 2 Agricultural Research Council (CRA-FRF), Forlì 47121, Italy; E-Mails: gianluca.baruzzi@entecra.it (G.B.); walther.faedi@entecra.it (W.F.)

**Keywords:** ribosome-inactivating proteins, strawberry, Dora cultivar, Record cultivar, plant stress, growth stage

## Abstract

Ribosome-inactivating proteins (RIPs), enzymes that are widely distributed in the plant kingdom, inhibit protein synthesis by depurinating rRNA and many other polynucleotidic substrates. Although RIPs show antiviral, antifungal, and insecticidal activities, their biological and physiological roles are not completely understood. Additionally, it has been described that RIP expression is augmented under stressful conditions. In this study, we evaluated protein synthesis inhibition activity in partially purified basic proteins (hereafter referred to as RIP activity) from tissue extracts of *Fragaria* × *ananassa* (strawberry) cultivars with low (Dora) and high (Record) tolerance to root pathogens and fructification stress. Association between the presence of RIP activity and the crop management (organic or integrated soil), growth stage (quiescence, flowering, and fructification), and exogenous stress (drought) were investigated. RIP activity was found in every tissue tested (roots, rhizomes, leaves, buds, flowers, and fruits) and under each tested condition. However, significant differences in RIP distribution were observed depending on the soil and growth stage, and an increase in RIP activity was found in the leaves of drought-stressed plants. These results suggest that RIP expression and activity could represent a response mechanism against biotic and abiotic stresses and could be a useful tool in selecting stress-resistant strawberry genotypes.

## 1. Introduction

Ribosome-inactivating proteins (RIPs) are plant *N*-glycosilases (EC 3.2.2.22) that depurinate the conserved α-sarcin loop of the large rRNA of prokaryotic and eukaryotic ribosomes, causing the irreversible arrest of protein synthesis. RIPs are classified into three types: type 1, consisting of a single polypeptide A chain of approximately 30 kDa; type 2, with an approximate molecular weight of 56–65 kDa in which an enzymatically active A chain is linked to a B chain with lectin properties and specificity for sugars exhibiting galactose-like structures; and type 3, composed of an *N*-terminal catalytic domain and an extended *C*-terminal domain of unknown function [1]. RIPs are widely distributed in the plant kingdom and are abundantly present in some plant families, particularly in the seeds of *Caryophyllaceae*, *Cucurbitaceae*, *Euphorbiaceae*, and *Phytolaccaceae* species [2,3]. RIP activity has also been identified in several edible plants, including some that are eaten raw by humans, though at levels that do not appear to be harmful [4]. In addition to the well-known toxic effect on ribosomes, some authors have reported that some RIPs exhibit other enzymatic activities. It has been shown *in vitro* that several RIPs can release adenine from different nucleic acid substrates, such as poly(A), mRNA, tRNA, and DNA [5], and some RIPs have also shown activity against poly(ADP-ribosyl)ated poly(ADP-ribose) polymerase [6]; accordingly, the denomination of polynucleotide:adenosine glycosilases (PNAG) has been proposed for this class of enzymes. Moreover, RIPs can act on viral nucleic acids [7] and autologous DNA [8].

The biological role of these proteins is not fully understood, and the reason why some plants accumulate RIPs in their tissues remains an unanswered question [9]. Nonetheless, the hypothesis of a defensive role is supported by several lines of experimental evidence. The expression of RIP genes can be regulated by biotic stress, including viral [10] or fungal [11,12] infections, and during plant senescence [13]. The proposed mechanisms for the RIP antimicrobial activity are quite controversial in literature; some authors identified the host ribosomes as the main target of endogenous RIPs, while others claimed that the defense mechanism may be exerted by direct interaction of RIP with invading pathogens (reviewed in [9]).

RIP expression has also frequently been reported to be increased by abiotic stress, such as mechanical injury [14,15], senescence, heat and osmotic stress [13], salinity, and drought [16–18]. Furthermore, the expression of RIP proteins is affected by some hormones, including jasmonic acid [12,14,19–21], abscisic acid [12,14,20], and gibberellic acid [22]. RIPs may be expressed in many isoforms in several tissues, including roots, leaves, flowers, fruits, and buds. Indeed, *Mirabilis jalapa* and *Saponaria officinalis* express RIP isoforms, with different patterns in different tissues [8,23], and four isoforms of Himalayan mistletoe RIP have been characterized from *Viscum album* [24].

In addition, RIP genes have been transfected into plants to increase their resistance to viruses, fungi [25–27], and insects [28,29].

In the present study, we investigated tissue extracts from whole plants of two different *Fragaria* × *ananassa* cultivars to evaluate the presence and variation in the RIP content in the partially purified basic protein fraction, as related to the plant life cycle and the abiotic stress. The soil-borne pathogen infection-resistant cultivar Record and susceptible Dora [30,31] were compared.

## 2. Results

**Figure 1:**
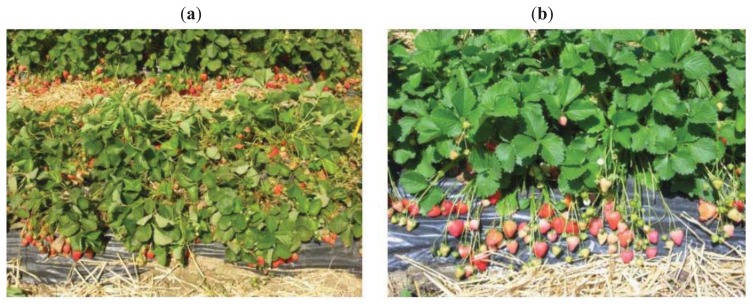
(**a**) Dora and (**b**) Record plants during fructification in fumigated soil (IPM). Dora is showing the first stress symptoms not evident in the Record plants.

The presence of RIP activity was investigated in the basic protein fractions of tissue extracts of two *Fragaria* × *ananassa* varieties with different levels of stress resistance: Dora and Record. Different cultivation conditions were analyzed to evaluate the influence of crop management techniques, organic culture, and fumigated soil, as a component of integrated pest management (IPM) (Figure 1). The plants were collected and analyzed during three periods corresponding to different growth stages, *i.e.*, quiescence, flowering, and fructification.

**Figure 2:**
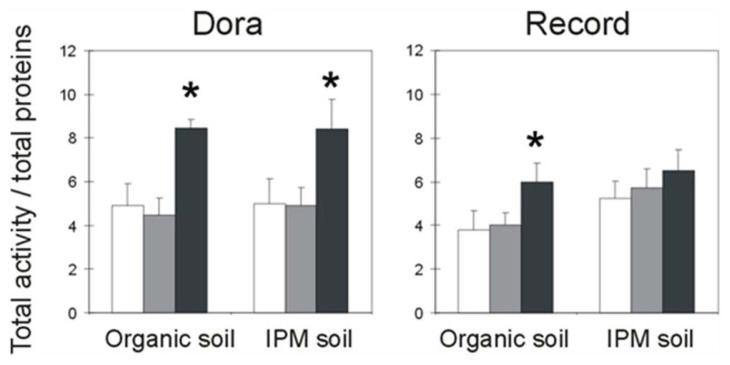
Comparison of ribosome-inactivating proteins (RIP) activity in whole *Fragaria × ananassa* plants during quiescence (white), flowering (grey), and fructification (black). The total activity was normalized to the entire basic protein fraction (mg). The protein concentration in the tested tissues ranged from 43.9 to 222.6 mg (quiescence), from 39.1 to 332.3 mg (flowering), and from 39.9 to 754.5 mg (fructification). The IC_50_ values ranged from 5 to 150 μg/mL. The results are the means ± standard deviation of three experiments, each performed in duplicate. The statistical analysis was performed using Student’s t test (confidence range 95%). The asterisks indicate statistical significance for the fructification period *versus* the quiescence and flowering periods (*p* < 0.05).

RIP activity was present in all the tested plants; the activity was expressed as the total activity normalized to the total protein content. Both cultivars showed a higher RIP activity during fructification than during quiescence and flowering periods. This enhancement was significant in Dora plants cultivated in organic or fumigated soil and in Record plants cultivated in organic soil but not in fumigated soil (Figure 2).

**Figure 3:**
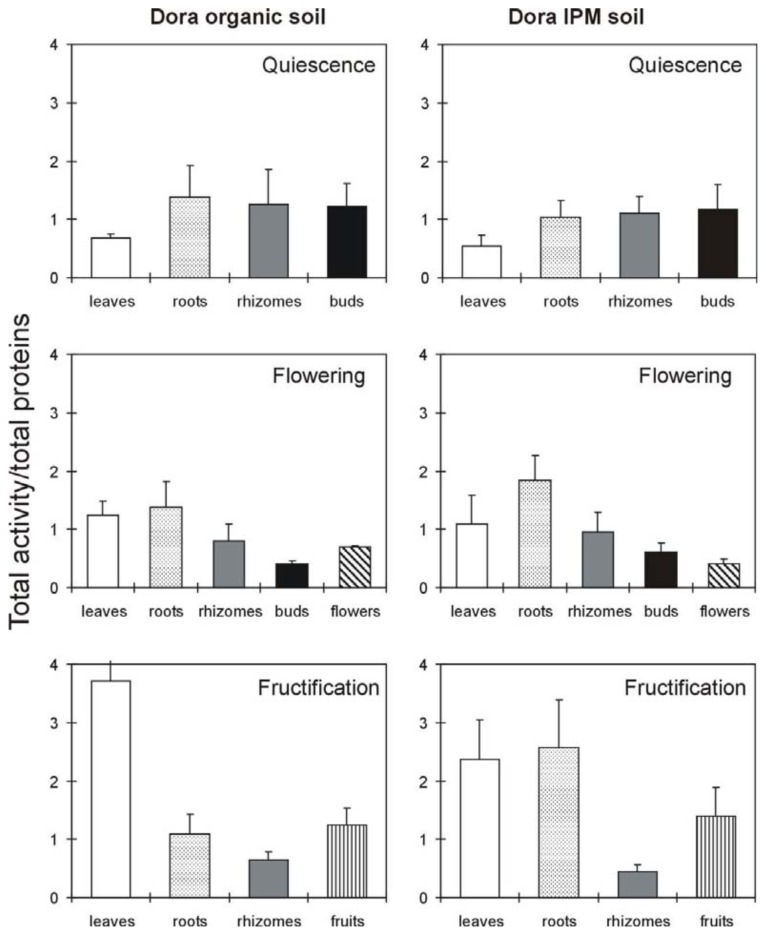
Distribution of total activity in different *Fragaria × ananassa* (cultivar Dora) tissues during various phases of the life cycle. The total activity was normalized to the total proteins (mg) present in each tissue. The IC_50_ values ranged from 5 to 143 μg/mL (supplementary file, Table S1). The values reported in the graphs are the total activity ± standard deviation. The results are the means ± standard deviation of three experiments, each performed in duplicate. The statistical analysis was performed using Student’s *t* test (confidence range 95%).

**Figure 4:**
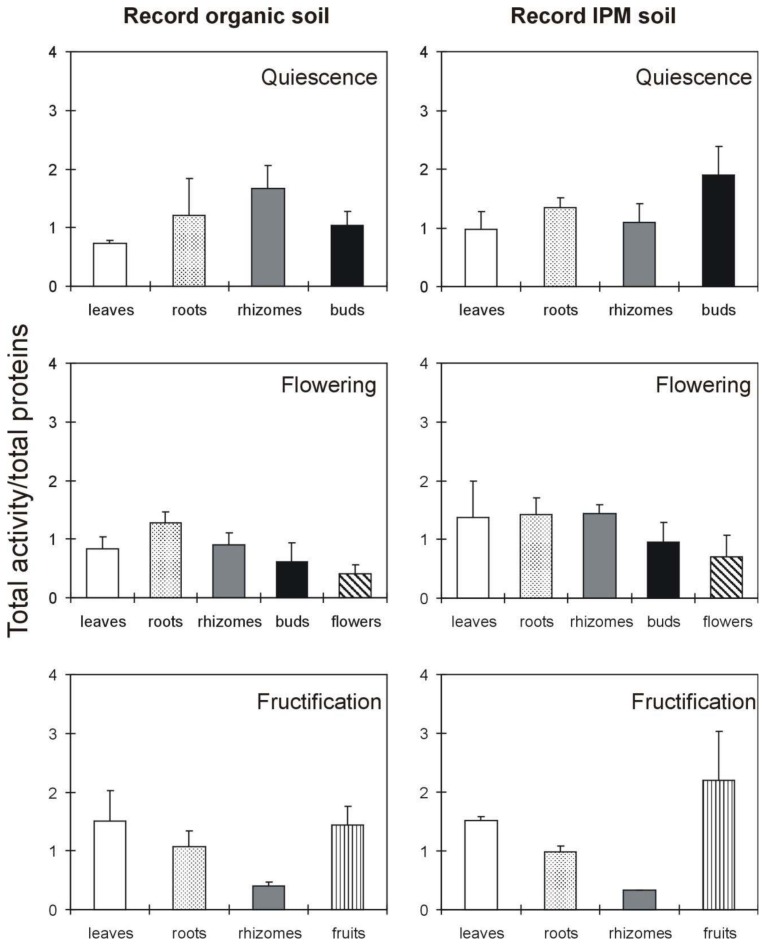
Distribution of total activity in different *Fragaria × ananassa* (cultivar Record) tissues during the various phases of the life cycle. The total activity was normalized to the total proteins (mg) present in each plant tissue. The IC_50_ values ranged from 9 to 150 μg/mL (supplementary file, Table S1). The values reported in the graphs are the total activity ± standard deviation. The results are the means ± standard deviation of three experiments, each performed in duplicate. The statistical analysis was performed using Student’s *t* test (confidence range 95%).

Samples were obtained from various tissues of the plant. In plants in quiescence stage the tissues analyzed were the roots, rhizomes, leaves, and buds. In plants in flower the roots, rhizomes, leaves, buds, and flowers were analyzed. In plants in fructification the roots, rhizomes, leaves and fruits (without a distinction between unripe and mature fruits) were analyzed (in this period buds and flowers were absent). RIP activity was present in each of the tested *Fragaria* × *ananassa* tissues, with concentrations that inhibited 50% of protein synthesis (IC_50_) ranging from 5 to 150 μg/mL. However, some interesting differences were found when the RIP activity in the various tissues was expressed as a percentage of the total activity of the entire plant (Figures 3 and 4). In the cultivar Dora, more susceptible to pathogens, the activity was present in the four evaluated tissues in more or less equivalent proportions during quiescence, being leaves the poorest (Figure 3). During flowering and fructification, there was a more unbalanced distribution of RIP activity, increasing in some tissues and decreasing in others. In particular, the RIP activity increased significantly in the leaves of Dora plants grown in organic soil during both flowering ( *p* = 0.02) and fructification ( *p* = 0.001), whereas the RIP activity of plants grown in the same conditions did not significantly vary in the roots and rhizomes during these stages, despite a tendency to diminish observable in rhizomes during fructification.

A different behavior was found in the Dora plants grown under IPM. RIP activity was significantly augmented in the leaves during fructification (*p* = 0.008) and in the roots during flowering (*p* = 0.039), remaining at a high level during fructification (*p* = 0.037). In contrast, the rhizomes showed a significant decrease during the fructification period (*p* = 0.014).

On the basis of the results for the Dora cultivar, the most important variations in RIP activity are as follows: (i) a strong increase in leaves during fructification both in organic conditions and under IPM, (ii) a high reduction in rhizomes during fructification under IPM (from 26% to 7%), and (iii) an increase in roots under IPM in flowering and in fructification.

Record, the more pathogen resistant cultivar, showed a different distribution of RIP activity compared to Dora, both with regard to the two cultivation conditions (organic or fumigated) and the three growth stages (Figure 4). During quiescence, the activity was mainly distributed in the storage tissues (rhizomes and roots) in the plants grown in organic soil, whereas a higher activity was present in the buds in plants grown under IPM. For plants grown in organic soil, the RIP activity remained constant in the roots during flowering and fructification, with significant decreases in the rhizomes both during flowering (*p* = 0.029) and fructification (*p* = 0.002). No significant enhancement was observed in the leaves during the flowering period, though a significant increase was detected during fructification (*p* = 0.041).

Therefore, the following tendency was observed for the Record plants grown under IPM: (i) the RIP activity remained constant during flowering in the roots and rhizomes but significantly decreased during fructification (*p* = 0.008 in roots and *p* = 0.005 in rhizomes); and (ii) a significant increase in the leaves was observed only during fructification (*p* = 0.039). On the basis of the results for the Record cultivar, the most important variations in RIP activity are as follows: (i) a large increase in leaves, and (ii) a reduction in rhizomes and roots during fructification, and (iii) a high RIP activity in fruits.

**Figure 5:**
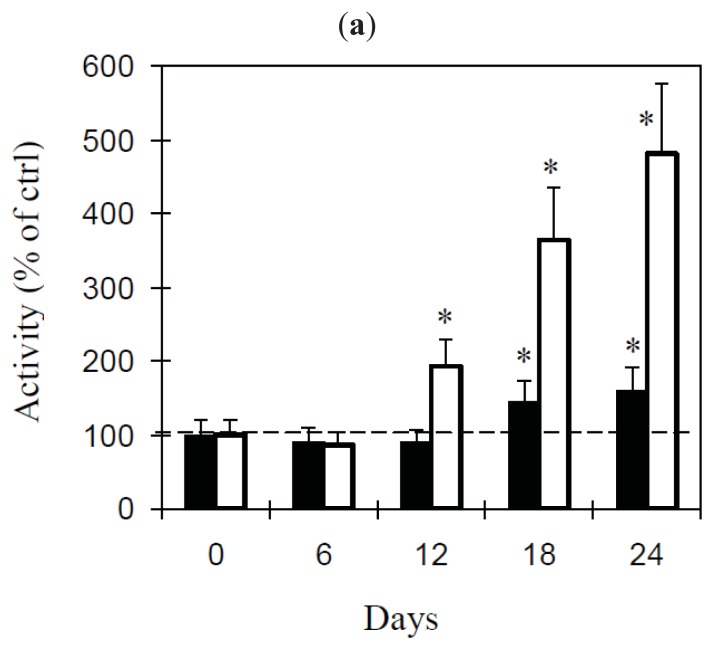

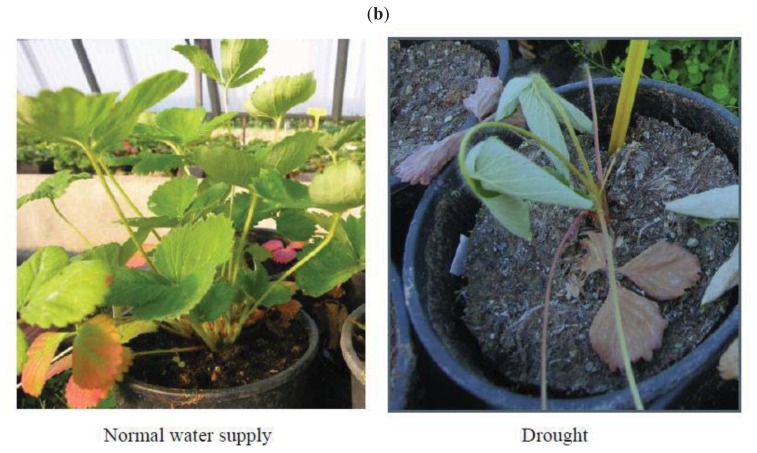
Effect of drought on Dora and Record plants. (**a**) Activity in Dora (black columns) and Record (white columns) stressed leaves. The activity (total activity, calculated as the specific activity per whole basic-fraction proteins, normalized to the total proteins of the crude extract) in stressed leaves is expressed as a percentage of that in the leaves of control plants grown under the same conditions and with a normal water supply. The IC_50_ values ranged from 11 to 100 μg/mL (supplementary file, Table S2). The results are the means of four experiments performed in duplicate. Standard deviation never exceeded 15%. The statistical analysis was performed using Student’s t test (confidence range 95%). The asterisks indicate statistical significance for the stressed plants in comparison with the plants at the start of the experiment (*p* < 0.0001); (**b**) Normal and stressed leaves of Record plants at day 18.

We also investigated the variations in Dora and Record RIP activity under drought stress conditions (Figure 5). In both varieties, the first signs of stress appeared in the leaves under drought conditions at 12 days after the water supply was discontinued, with the plants presenting the wilting of young leaves. The effects of stress continued at 18 days, a time when the young and mid-aged leaves were wilted and continued up to 24 days when all the leaves were wilted. All the plants died after 27–30 days. Despite this apparently similar fate in both Dora and Record, some important differences appeared when the RIP activity was analyzed. In the leaves of Dora, a slight increase in RIP activity was observed, starting from 18 days, with values approximately 1.5-fold higher than the control. In Record, an earlier and higher increase in RIP activity was observed, with values of approximately 2-fold after 12 days and approximately 5-fold after 24 days with respect to the control plants (Figure 5a).

## 3. Discussion

To date, no systematic study has performed on the variation of RIP distribution in the tissues of edible plants grown in the field.

We chose *Fragaria × ananassa* (strawberry) for our experiments because of its rapid growth cycle (approximately 3 months) and of the availability of many varieties with different degrees of resistance to climatic conditions and pests. The plant tissues analyzed in our study were obtained from two different varieties of *Fragaria × ananassa*, Dora and Record, which are characterized by different sensitivities to adverse weather conditions and pathogens, being Record the more resistant to pathogens and fructification stress [30,31]. Furthermore, the plants were grown in soils subjected to two different treatments. The organic soil involved fertilization with cattle manure, providing all the nutrients required for plant growth but not protecting the plants against pathogens. The soil fumigation (IPM) involved the treatment with a mixture of chloropicrin and 1,3-dichloropropene, eradicating most of the soil pathogens.

In this study, the growth stage of *Fragaria × ananassa* plants and the RIP activity distribution were analyzed during three periods of plant development: quiescence, flowering, and fructification. The partially purified basic fractions from various tissues were evaluated on a protein synthesis cell-free system. Based on the well-known properties of RIPs, the observed inhibition of protein synthesis in the experimental conditions of the present work is very likely due to RIP presence. The analysis of the presence of RIP in various plant tissues showed a wide distribution. The RIP activity was distributed in all the examined tissues in both cultivars, with IC_50_ values ranging from approximately 5 to 150 μg/mL. These differences could be the result of the expression of different isoforms with different activities, as previously observed in other plant species [8,23].

The Dora plants showed a higher RIP activity during fructification than during the quiescence and flowering periods, independent of the soil treatment; this result could be related to the plant stress that is associated with fruit production, which is the most intense phase of the plant life cycle. Record plants showed an increase in RIP activity during the fructification period that was significant only when the plants were grown in organic soil. These results support the hypothesis that the RIP level increases under stress conditions [16] and/or during plant senescence [13]. The more resistant Record cultivar was exposed to lower stress and exhibited an insignificant increase in RIP activity when grown under IPM, most likely due to the low level of pathogens in this soil.

The analysis of the distribution of RIP activity in Dora and Record plants revealed a tendency to increase in leaves and a tendency to reduce in rhizomes during flowering and fructification both in organic and in fumigated soil. However these variations are not always significant. RIP activity in the Dora plants strong increases in leaves during fructification and markedly reduces in rhizomes both in organic and fumigated soil. The high RIP activity observed in Dora leaves could be explained by the low resistance of the Dora cultivar to pathogens. Moreover, in fruiting the high level of RIP activity in Dora leaves should be considered as a stress marker due to senescence [13]. In fact, Dora plants during fructification show a collapsed foliage. The Record cultivar exhibited an increase in leaf RIP activity during fructification, though to a lesser extent than Dora. Both cultivars showed a medium/high fruit RIP activity. It is well known that strawberry fruits can be highly allergenic. As RIPs are very immunogenic and can cause allergic reactions in exposed individuals [32], it can be hypothesized a RIP contribute to the allergenic reaction to strawberry fruits. Studies confirming these observations could have an important impact on the safety of strawberry consumption, leading to the selection of cultivars with a low content of RIPs in edible tissues.

It was previously reported that the RIP activity could increase after drought conditions [13,17,18], and some authors proposed it as a defense mechanism. This has been proposed to be due to the reorganization of protein metabolism through the RIP inhibition of protein synthesis and/or to the SOD activity, reported for some RIPs [4,18,33]. In this study, we evaluated the association between RIP activity and drought stress in the Dora and Record cultivars. All the stressed plants showed a strong increase in leaf RIP activity, but considerable differences between the two varieties were noted: significant differences between stressed and non-stressed Dora plants were found only after 18 days, whereas Record showed significant differences after 12 days. Moreover, the drought-stressed ‘Record’ plants showed higher RIP activity in the leaves than in the similarly stressed Dora plants, suggesting that in the first period ‘Dora’ may be more resistant to drought than Record. However, when the stress conditions go on, the high resistance of Dora could be not more sufficient to preserve the plant from death.

## 4. Experimental Section

### 4.1. Materials

The materials for low-pressure chromatography, including the calibrating substances, were obtained from GE Healthcare (Milan, Italy), and Amicon (Beverly, MA, USA). The fresh plant tissues were homogenized using an Ultra-turrax apparatus (Ika, Staufen, Germany). A cell-free protein synthesis inhibition assay was performed with l-[4,5-^3^H] leucine from Amersham Biosciences (Buckinghamshire, UK), and the incorporation of radioactivity was evaluated using a β-counter and Ready-Gel from Beckman Instruments (Fullerton, CA, USA). The total protein concentration was assessed using a UVIKON 860 spectrophotometer from Kontron Instruments (Everett, MA, USA). All of the chemical reagents used for preparation of crude extracts and basic protein fraction and for determination of translation inhibitory activity were from Sigma-Aldrich (St. Louis, MO, USA).

### 4.2. Plant Materials and Growth Conditions under Normal and Stress Treatments

The study was performed in the Cesena area (Po Valley) using the Italian strawberry cultivars Dora and Record in two adjacent fields on the same farm under identical soil conditions to compare performance under IPM and organic cultivation system regimes. Dora is a variety having as its main limit the strong susceptibility to collapse because of the black root complex causing wilting particularly severe in old soils or non-fumigated soils. On the other hand, Record does not present this susceptibility and could be cultivated in all types of soil successfully, especially in non-fumigated ones. In fumigated soils, indeed, the Record plant becomes too vigorous and this does not allow an optimal level of production.

The trials were managed according to the recommendations for annual open-field commercial crops published by Emilia-Romagna Region. Organic cultivation system received 4 t/ha of organic compost at pre-planting and 75 kg/ha organic N during the cropping cycle through 5 fertigations. The IPM system used soil fumigation at pre-planting with a mixture of chloropicrin (30 g/m^2^) and 1,3-dichloropropene (25 g/m^2^) and 10 kg/ha of mineral N supplied by fertigation. A completely randomized block design with four replications of single plots of 20 cold-stored plants per cultivar, planted in the end of July, was adopted in both cultivation systems.

### 4.3. Water Stress

The two cultivars “Record” and “Dora” were grown under optimized controlled-climate conditions in a screen house for thirty days. The initiation of two treatments, drought and watered, was considered day 0, and samples of 2 leaves from 4 pots per treatment were collected at days 6 (no stress symptoms), 12 (young leaves wilted), 18 (young and mid-aged leaves wilted), and 24 (all leaves wilted).

### 4.4. Preparation of Crude Extracts and Basic Protein Fraction

Fresh plant materials (roots, rhizomes, leaves, buds, flowers, and fruits) from the Dora and Record cultivars were frozen in liquid nitrogen, ground in a mortar, and homogenized using an Ultraturrax apparatus in cold phosphate-buffered saline (0.14 M NaCl and 5 mM sodium phosphate buffer, pH 7.5) and incubated overnight at 4 °C on a magnetic stirrer. The homogenate was then filtered through cheesecloth, adjusted to pH 4.0 with glacial acetic acid, and clarified by centrifugation 18,000× *g* for 40 min at 4 °C. The supernatant was applied to an S-Sepharose Fast Flow column (5 × 1 cm) equilibrated with 10 mM sodium acetate (pH 4.5) at room temperature. The column was extensively washed with the equilibration buffer, and the proteins were eluted with 5 mM sodium phosphate buffer (pH 7.0). The crude extract and eluted protein concentrations were assessed spectrophotometrically applying the Kalb and Bernlohr method. The activity was analyzed immediately after protein determination.

### 4.5. Determination of Translation Inhibitory Activity

The inhibitory effect of the basic protein fractions on translation in a cell-free protein synthesis system (rabbit reticulocyte lysate) was estimated as described previously [34]. The reaction mixtures contained, in a final volume of 62.5 μL, 10 mM Tris-HCl (pH 7.4), 100 mM ammonium acetate, 2 mM magnesium acetate, 1 mM ATP, 0.2 mM GTP, 15 mM phosphocreatine, 3 μg creatine kinase, 0.05 mM amino acids (minus leucine), 89 nCi l-[4,5-^3^H]leucine, and 25 μL rabbit reticulocyte lysate. The reaction was incubated at 28 °C for 5 min. The reaction was arrested with 1 mL of 0.1 M KOH, and two drops of H_2_O_2_ and 1 mL of 10% (*w*/*v*) of trichloroacetic acid were added. Precipitated proteins were collected on glass-fiber disks, put in vials with 5 mL of Ready Gel and the radioactivity incorporated into protein was measured with a β-counter. Each experiment was performed in duplicate, and the results are expressed as the mean of three different experiments. The concentration giving 50% inhibition (IC_50_) was calculated by a linear-regression analysis. Total activity was calculated as the specific activity per whole basic-fraction proteins (mg) normalized to the total proteins of the crude extract. Specific activity is expressed as units (U) per mg of protein where one U is the amount of proteins (in μg) that inhibits 50% protein synthesis in 1 mL of reaction mixture.

### 4.6. Statistical Analysis

The experimental data are expressed as the mean ± standard deviation. The results were statistically evaluated using Student’s *t* test (confidence range 95%). The statistical analysis was performed using XLSTAT-Pro software, version 6.1.9 (Addinsoft 2003, New York, NY, USA).

## 5. Conclusions

The results presented in this study demonstrate an increased RIP activity in strawberry plants under reproductive, biotic and abiotic (drought) stresses. The association found between RIP expression and biotic and abiotic stresses necessitate the elucidation of RIP involvement in the stress response pathway. The knowledge of the role of these enzymes could be important for the selection of cultivars that are more resistant to adverse environmental conditions.

## Supplementary Information
