# Supplementary material for: Protein Synthesis Inhibition Activity by Strawberry Tissue Protein Extracts during Plant Life Cycle and under Biotic and Abiotic Stresses
Source: Int J Mol Sci. 2013 Jul 25;14(8):15532–45. doi: 10.3390/ijms140815532 (PMC3759871; doi:10.3390/ijms140815532)
Supplement: Supplementary file 1 [file ijms-14-15532-s001.pdf]

Supplementary Information

Table S1. IC_{50} used for the calculation of the activities reported in Figures 3 and 4.

IC_{50} (μg/mL) [a]					
**Growth srages**	**Tissues**	**Dora**		**Record**	
		**Organic soil**	**IPM**	**Organic soil**	**IPM**
Quiescence	leaves	17.5	16.2	10.4	12.9
	roots	19.6	25.0	19.6	19.6
	rhizomes	51.0	54.6	56.2	69.8
	buds	21.6	12.7	21.5	13.0
Flowering	leaves	11.0	22.0	19.4	23.3
	roots	40.1	46.8	34.2	26.9
	rhizomes	72.9	78.2	63.8	60.4
	buds	23.3	26.9	16.8	16.6
	flowers	39.7	31.9	28.0	33.2
Fructification	leaves	39.3	38.6	29.1	33.9
	roots	47.1	43.2	35.3	51.3
	rhizomes	97.5	139.6	148.8	147.0
	fruits	41.8	50.6	37.8	35.7

[a] The IC_{50} values reported in tables are the mean of duplicate experiments for each tissue × 4 individual plants.

Table S2. IC_{50} used for the calculation of the activities reported in Figure 5.

IC_{50} (μg/mL) [a]					
	**Day**	**Dora**		**Record**	
		**Normal**	**Drought**	**Normal**	**Drought**
	0	11.4	31.5	40.3	37.5
	6	32.7	35.8	58.6	47.9
	12	58.1	27.8	49.8	42.2
	18	47.7	62.0	95.5	42.6
	24	44.2	50.2	98.8	58.2

[a] The IC_{50} values reported in tables are the mean of duplicate experiments for each tissue × 4 individual plants.

© 2013 by the authors; licensee MDPI, Basel, Switzerland. This article is an open access article distributed under the terms and conditions of the Creative Commons Attribution license (http://creativecommons.org/licenses/by/3.0/).
